# Quantitative Analysis of Situation Awareness During Autonomous Vehicle Handover on the Da Vinci Research Kit

**DOI:** 10.3390/s25113514

**Published:** 2025-06-02

**Authors:** Tamás Levendovics, Dániel A. Drexler, Nikita Ukhrenkov, Árpád Takács, Tamás Haidegger

**Affiliations:** 1 Antal Bejczy Center for Intelligent Robotics, University Research and Innovation Center, Obuda University, Bécsi út 96/B, 1034 Budapest, Hungary; tamas.levendovics@irob.uni-obuda.hu (T.L.); drexler.daniel@nik.uni-obuda.hu (D.A.D.); nikita.ukhrenkov@irob.uni-obuda.hu (N.U.);; 2 Doctoral School of Applied Informatics and Applied Mathematics, Obuda University, Bécsi út 96/B, 1034 Budapest, Hungary; 3 Institute of Biomatics and Applied Artificial Intelligence, John von Neumann Faculty of Informatics, Obuda University, Bécsi út 96/B, 1034 Budapest, Hungary; 4 Austrian Center for Medical Innovation and Technology (ACMIT), Viktor-Kaplan-Straße 2/1, 2700 Wiener Neustadt, Austria; 5 School of Computing, Queen’s University, Kingston, ON K7L 2N8, Canada

**Keywords:** autonomous vehicle safety, self-driving, driving simulator, hand-over, situation awareness

## Abstract

The current trends in the research and development of self-driving technology aim for Level 3+ autonomy, where the vehicle controls both lateral and longitudinal motions of the dynamic driving task, while the driver is permitted to divert their attention, as long as they are able to react properly to a handover request initiated by the vehicle. At this level of autonomy, situation awareness of the human driver has become one of the most important metrics of safety. This paper presents the results of a user study to evaluate handover performance at Level 3 autonomy. The study investigates whether the level of situation awareness during critical handover situations has a direct impact on task performance, with higher situation awareness expected to lead to better outcomes during emergency interventions. The study is performed in a simulated environment, using the CARLA driving simulator and the master console of the da Vinci Surgical System. The test subjects were asked to answer the questions of a questionnaire during the experiment; the answers for those questions and the measured control signals were analyzed to gain further knowledge on the safety of the handover process.

## 1. Introduction

**Figure 1:**
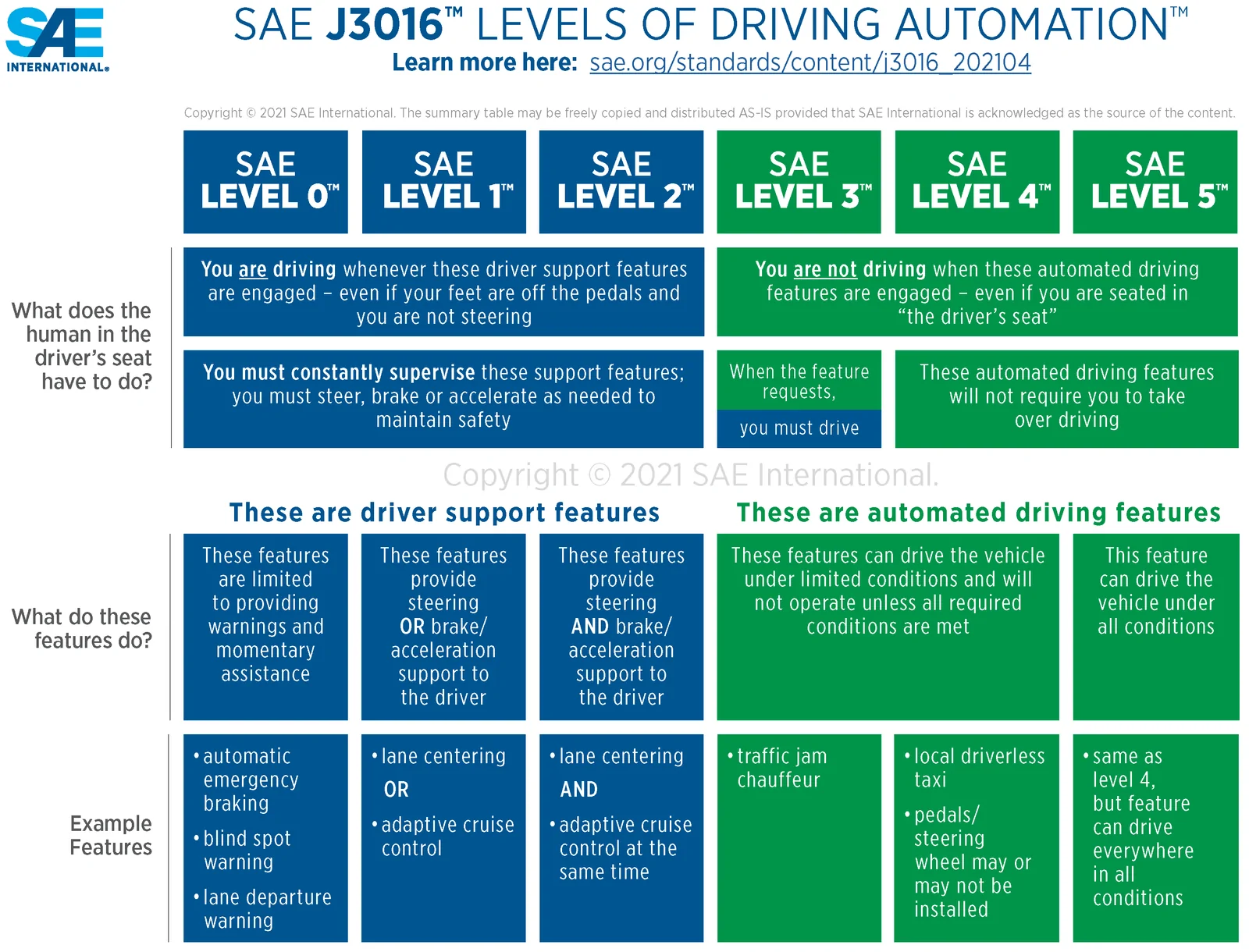
Level of autonomy (LoA) concept for automated vehicles introduced by the Society of Automotive Engineers International [7].

In the past few years, automotive technologies have had a huge focus in research and development [1]. Fully automated vehicles are not out of the question in the near future; however, there are still large milestones to achieve in development and safety [2,3]. Driving involves a wide range of skills—such as lane keeping, hazard anticipation, decision making in intersections, and reacting to unexpected events—that vary depending on the situation [4,5,6]. For example, highway cruising primarily requires sustained attention and lane discipline, while urban driving demands continuous perception, negotiation with pedestrians, and short-term planning. Parking involves spatial awareness and precise control, while emergency response requires fast reflexes and situational assessment. As automation increases, different subsets of these skills are delegated to the system, but others must still be retained by the human. Therefore, new approaches are needed to manage shared control, and it remains essential to keep the human partially involved. The necessary skills depend on the level of automation. In automated driving technologies, there is a widely accepted classification of autonomy, which was introduced by the Society of Automotive Engineers (SAE) International (Figure 1), and is used in other research areas as well [7,8]:

- **LoA 0**: No automation. The vehicle is only permitted to send warning signals to the driver, it cannot interfere any of the controls. The human driver is responsible for controlling the vehicle in all aspects.
- **LoA 1**: Driver assistance. The vehicle is allowed to control either steering or acceleration in cooperation with the human driver.
- **LoA 2**: Partial automation. The vehicle performs complex actions by controlling both steering and acceleration in limited use-cases. The constant monitoring of the environment by the human driver is still required.
- **LoA 3**: Conditional automation. The vehicle is prepared for the dynamic driving task by limited perception and decision-making abilities. The human driver is allowed to divert its attention, in such a manner that they are able to take control back at any time if a fall-back event occurs.
- **LoA 4**: High automation. The vehicle is equipped to perform the dynamical driving task in pre-defined driving modes. No real-time human–machine interaction is required, as the vehicle is able to move to a safe state from an emergency, among all possible conditions. In this safe state, the human driver could take over control.
- **LoA 5**: Full automation. The vehicle is able to accomplish the dynamic driving task in all the driving modes, regardless of the environment conditions.

The watershed step is in between LoA 2 and LoA 3, not just in the technological terms, but in safety as well. LoA 2 still belongs to the domain of well-known advanced driver assistance systems (ADAS), which means that the user is fully responsible for the driving and for the possible damages. In the case of LoA 3, the driver is partly responsible for the decision making: if the system gives a signal indicating it cannot handle the situation, the driver has to continue the procedure immediately and make decisions. This shift places new demands on the driver’s cognitive readiness—particularly in critical situations. One of the key concepts in assessing this readiness is situation awareness (SA), which refers to the human operator’s perception, comprehension, and projection of environmental elements [9]. In LoA 3, the risk of reduced SA is particularly pronounced, as drivers may become disengaged during passive monitoring, leading to slower or inappropriate reactions during emergency handover events [10,11].

In this paper, we introduce an experimental study to examine situation awareness in self-driving technologies under critical conditions. To objectively measure handover, we created a novel system architecture, using the master console of the da Vinci Surgical System (Intuitive Surgical Inc., Sunnyvale, CA, USA) as Human–Machine Interface (HMI) alongside the CARLA Simulator [12,13]. The da Vinci system, which is a master–slave teleoperation robot, is capable of providing the limited view of the environment, which is critical in SA. While our earlier work introduced a preliminary version of this setup with a small sample size and limited analysis [14], the current study expands on that foundation in several key ways. Based on insights from the initial experiments, we refined the scenario design—removing weather-based variations such as heavy rain, which showed no significant effect. Additionally, we increased the participant pool from 7 to 15 licensed drivers and conducted a more comprehensive evaluation. We model the handover process under emergency with different situations, such as a true/false alarm by the automated system, and a car arriving and no car arriving from the other lane. The experiments were performed by 15 subjects (everyone owns a driving license). The situation awareness is measured with a combination of freeze probe technique, self-rating, and performance measures [15]. We asked the participants to fill out a questionnaire after each scenario, we requested information about the environment, and asked them to rate themselves and use other information from the simulator (e.g., collisions, time between handover signal and first modification of control inputs) to rate the performance. We seek the answer to the question of whether we can measure the SA of the participants, and whether we can decide the level of SA and its effect on the performance during an emergency situation. We hypothesize that the level of SA during critical handover situations directly affects task performance—participants with higher SA will demonstrate better performance during emergency interventions.

## 2. Related Work

The progression of automated driving technologies has led to extensive research into human–automation interaction, particularly around safety-critical aspects, such as SA, ethical responsibility, and system design. This section presents an overview of the relevant literature spanning from the definition of automation levels and driver behavior and finally to methods for evaluating SA in the Internet of Cars (IoC) environments.

The widely adopted classification system by the Society of Automotive Engineers (SAE) [7] divides vehicle autonomy into six levels (LoA 0 to 5), providing a common framework for researchers and developers, cross-fertilizing other domains of robotics, emphasizing the necessity for clear role allocation and control responsibility [8].

The transition from LoA 2 to LoA 3 is of particular interest due to the shift in operational responsibility, and the introduction of conditional automation. While LoA 2 remains within the scope of advanced driver assistance systems (ADAS), LoA 3 introduces limited decision-making capabilities and conditional control transfer, requiring human drivers to be ready to intervene at any moment [2,3]. The main issue with LoA 3 is that the essential functions of driving are automated, and because of this, the driver can easily be distracted—which can be crucial under critical conditions. Studies show that the human mind is not effective in long inactive monitoring tasks, and usually over-trusts in the automated system [16,17,18,19,20,21,22]. Because of these reasons, in LoA 3, safety considerations are indispensable along with systematic design methods to ensure the safety and integrity of such systems [23].

The notion of SA is central to understanding driver effectiveness in partially automated systems. Endsley’s seminal model [5,9] divides SA into three levels:

- **Level 1 SA**—perception of the environment;
- **Level 2 SA**—comprehension of the current situation;
- **Level 3 SA**—projection of future status.

This framework has been widely adopted in domains requiring dynamic human–machine collaboration, including aviation [24] and autonomous driving [15,25].

Matthews et al. describe the following components of SA [26]:

- *Spatial awareness*—knowledge of object locations;
- *Identity awareness*—knowledge of salient items;
- *Temporal awareness*—knowledge of the dynamic states;
- *Goal awareness*—knowledge of the maneuvering plan;
- *System awareness*—knowledge of the environment.

Accurately measuring SA is essential for understanding how drivers perceive, comprehend, and respond to dynamic handover scenarios in partially automated systems. One of the primary methods employed is the freeze probe technique, a widely recognized approach for assessing real-time cognitive processing. Originally developed as part of the situation awareness global assessment technique (SAGAT) [27], the freeze probe method involves interrupting a task at predetermined points to assess the participant’s awareness of critical situational elements. By temporarily halting the simulation and requiring participants to recall specific environmental details, this technique provides a direct measure of SA at the moment of handover. Research has demonstrated that the freeze probe approach mitigates the limitations of post-hoc recall biases, offering a more reliable indicator of real-time cognitive processing [28].

Self-rating scales are also employed commonly to capture participants’ subjective perceptions of their SA during the handover scenarios. Self-assessment techniques such as the situational awareness rating technique (SART) [29] have been widely used in the literature to evaluate perceived cognitive workload and situational comprehension. While subjective measures are inherently influenced by individual biases, these provide valuable insight into participants’ confidence in their understanding of the situation, as well as their perceived ability to respond effectively.

Several studies have employed objective performance metrics to assess SA, focusing on behavioral indicators such as reaction time, response accuracy, and the quality of decision making during simulated handover scenarios [16]. Task performance is often considered an indirect measure of SA, as individuals with greater awareness typically respond more quickly and effectively to dynamic situations [9,30]. However, performance outcomes alone may not fully reflect a participant’s SA, as effective responses can sometimes result from compensatory behavior rather than an accurate mental model of the situation. Research has shown that self-rating scales can serve as a useful complement to objective measures, particularly when combined with performance-based assessments [31,32,33,34].

**Figure 2:**
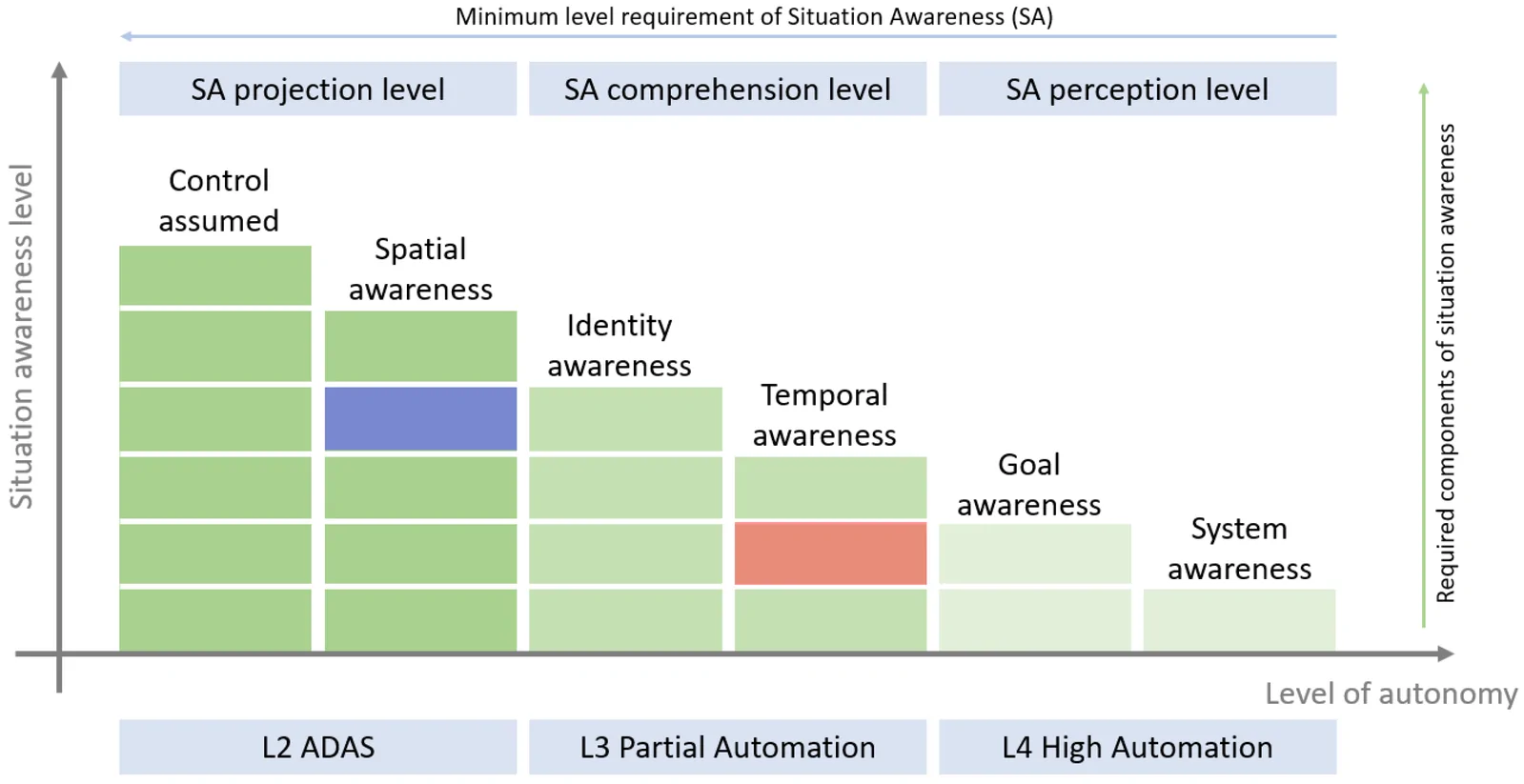
Hierarchical representation of situation awareness (SA) blocks in automotive solutions. For each level of autonomy, the quantitative metrics must fulfill the requirements for each block. The red-highlighted block represents the SA metrics related to LoA 3, specifically focusing on the comprehension of dynamic states. In contrast, the blue-highlighted block reflects the driver’s ability to understand the spatial structure of the environment while operating under a LoA 2 driver assistance system.

A systematic, quantitative assessment of SA is essential for the safe deployment of automated driving systems. While existing techniques like the freeze-probe method, self-rating scales, and performance metrics offer valuable insights, there is still no widely accepted, standardized approach to fully quantify SA across different levels of automation. As automation increases from LoA 2 to LoA 4, the demands on the human driver evolve, with the system taking on more responsibility for certain SA components. To better define and evaluate these changing requirements, a hierarchical framework has been proposed, linking SA to both environmental awareness (SA levels) and knowledge-based components [15]. This framework provides a way to measure SA at different levels of automation, offering a structured approach for integrating autonomous features while considering the cognitive load on the driver (Figure 2). Empirical testing of these metrics remains essential to refine the understanding of SA and to develop future automotive safety standards [35].

In the literature on SA in self-driving technologies, researchers have employed a variety of systems to assess driver awareness during critical conditions. For instance, studies have utilized advanced driving simulators equipped with high-fidelity visual and haptic feedback to simulate complex driving scenarios, enabling the assessment of driver responses and SA in controlled environments [16,30]. Additionally, some research has been conducted using real-world testing with instrumented vehicles, allowing for the collection of empirical data on driver behavior and SA during actual driving conditions [19].

Recent studies on SA in self-driving vehicles emphasize the complexities of human–automation interaction, particularly when transitioning between different levels of automation. For example, Lee et al. [36] demonstrated that sharing vehicle situation awareness with drivers in urban environments can significantly reduce driver-initiated overrides, thus enhancing safety and minimizing errors. This is especially crucial in partially automated systems, where drivers may lose SA due to the system handling most driving tasks. Other works, such as those by Chen et al. [37] and Salmon et al. [38], underscore the challenges in evaluating and measuring SA in these environments, as the cognitive load on drivers increases with the automation level.

The role of SA varies across different levels of automation. In the cases of LoA 0, 1, and 2, SA is obviously important: the driver has to constantly monitor and understand the environment, and estimate the future. The SA aspect of LoA 2 appears in aviation automation: the pilot has to handle at least one function of the cruising, which can help not to lose SA [5,24]. In LoA 3, SA is more special: while the autonomous functions are working well, the driver can easily lose SA, and when it is necessary, they cannot react well or acceptably fast [10,11,36,38,39]. This situation, when the driver has to take back the control, is called *handover*, and the necessary time for it is called *takeover* [11]. In non-critical scenarios—such as planned exits from highways or transitions without immediate hazards—takeover times vary significantly, typically ranging from 1.9 to 25.7 s, depending on driver engagement and presence of secondary tasks [6]. These situations lack urgent time pressure, and the wide variance in response times highlights the need for accommodating individual differences. In contrast, critical situations with imminent hazards often require faster reactions, yet may produce delayed responses due to increased cognitive load.

The role of SA becomes particularly crucial in LoA 3 systems, where drivers may face difficulties maintaining appropriate levels of awareness during periods of low engagement, especially in high-stakes situations like critical takeovers [16,30]. Studies on a Tesla crash [18] further reinforce this point, illustrating how a lack of adequate SA contributed to the fatal accident. These findings demonstrate the critical need for innovative approaches to both enhance and evaluate SA, ensuring that drivers remain adequately prepared to intervene when required, especially as automation systems progress to higher levels of autonomy.

## 3. Experimental Setup

**Figure 3:**
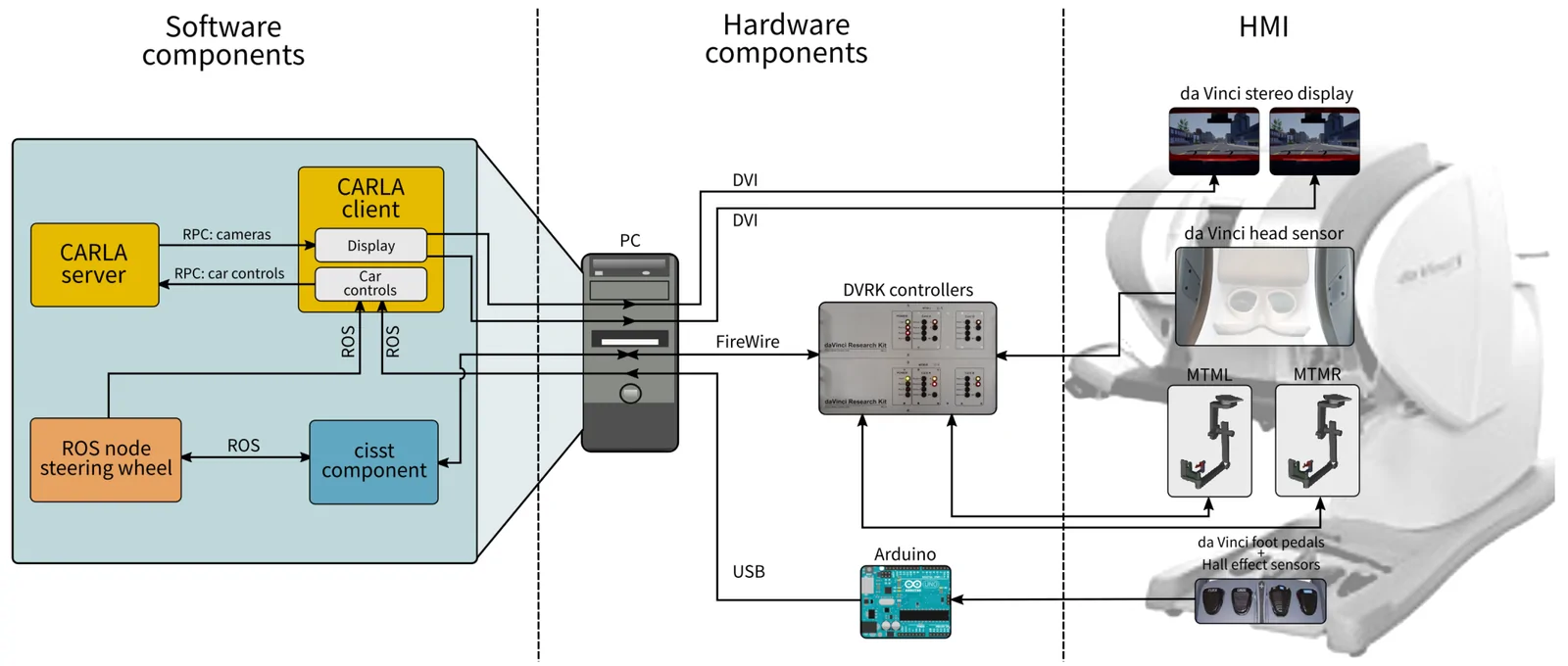
Experimental setup with the da Vinci Surgical System to examine situational awareness under critical conditions. The da Vinci master provides the display, the head sensor, the wheel, and the pedals to imitate a driving environment, and the setup is linked to the CARLA driving simulator with ROS components.

**Figure 4:**
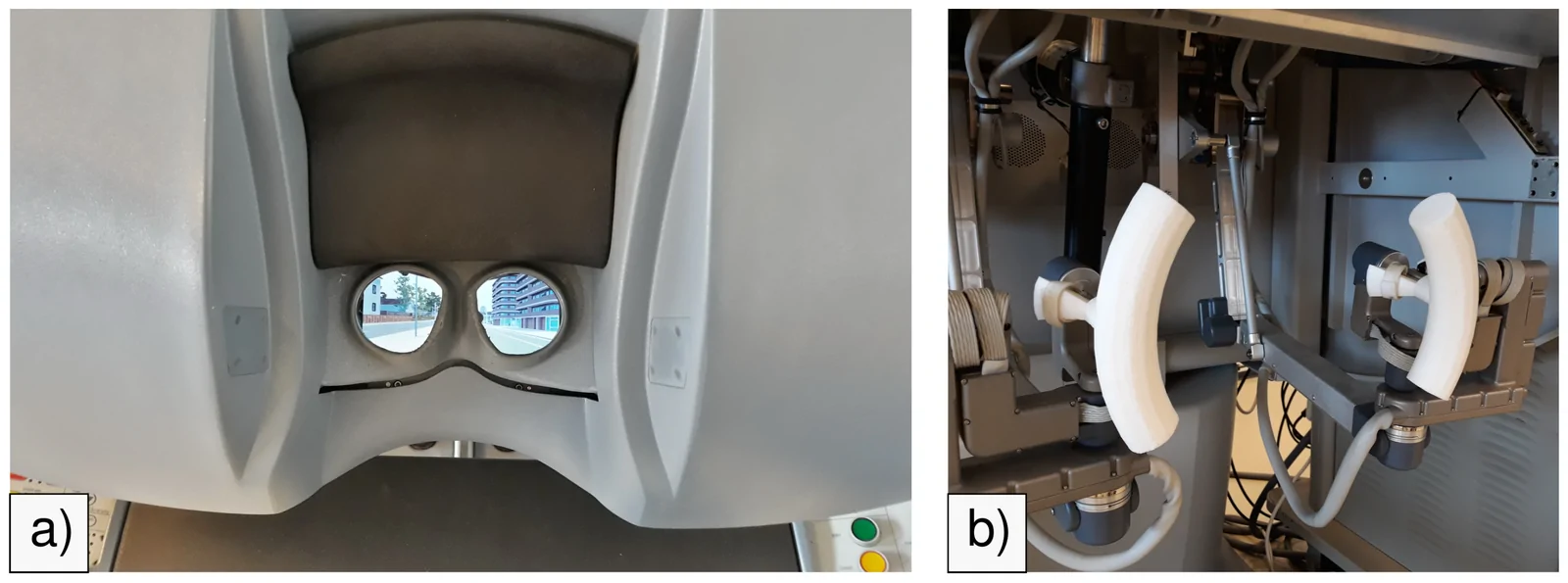
The da Vinci master console modified for SA measurements in self-driving handover situations. (**a**) The da Vinci master console’s stereo display with an integrated CARLA car simulator setup; (**b**) the da Vinci Surgical System master arms amended with 3D printed wheel segments to imitate a steering wheel.

The da Vinci Surgical System was developed for teleoperated robot-assisted minimally invasive surgery (RAMIS), and because of its human–machine interface, it is sufficiently versatile to be adapted for self-driving handover experiments (Figure 3) [40]. The da Vinci system involves a master and a slave robotic side, where the master is manipulated by a human operator. In the master console, the da Vinci system provides a fixed head position, where the operator can only see the monitor, but cannot see the environment around him, and vice versa—when his head is not inside the required area, he cannot see the monitors (Figure 4a). Furthermore, the da Vinci has a head sensor to detect whether the user is looking into the monitors or not (in the case of RAMIS it is a critical factor). With the fixed head position and built-in head sensor, attention can be monitored. The master console’s design also aligns well with driving simulations, as its two manipulator arms (master tool manipulators, MTMs) can be easily adapted to simulate the steering wheel and foot pedals.

In this research, we amended MTMs with a steering wheel, and the 3D printed wheel parts are attachable to the MTMs (Figure 4b). This solution provides quick prototyping, and furthermore, a reproducible system for other da Vinci research laboratories. The research software of the da Vinci (see later) provides built-in impedance control mode for the master arms. Thus, we can imitate the motion of a steering wheel [41]. The result of this solution is that the arms can only move along a circular path.

The da Vinci pedals were originally created for binary operations, such as controlling energy devices, or to manipulate the endoscope holder arm during surgery. For our research purposes, we had to modify the pedals to ensure continuous state reading. For this, Hall effect sensors and magnets were used for the accelerator and brake pedals. The sensors were connected to an Arduino board (Arduino Co., Somerville, MA, USA) [42], which read the sensor values.

The core software components of the experimental setup were the da Vinci Research Kit (DVRK) [12] and the CARLA Simulator (http://carla.org/ (accessed on 29 May 2025)). DVRK is an open-source hardware and software set, which provides complete read and write access to the da Vinci arms. CARLA is an open-source driving simulator, and in this study it was interfaced to the da Vinci master. To link the components of the system, Robot Operating System (ROS) (https://www.ros.org/ (accessed on 29 May 2025)) was used, which is a well-known library for robotic research [43]. DVRK, CARLA, and Arduino support ROS communication [13].

The simulation core ran inside the CARLA server. A CARLA client was ran by the server, which was implemented in Python, and used remote procedure calls to forward the steering angle of the wheel and the pedal values received via ROS to the server. The client defines the cameras for stereo vision as well (Figure 3). The pedal information is read by the Arduino, and then published into a ROS topic. Another ROS node performs the settings of the impedance control gains, and then it publishes the steering angle into a ROS topic towards the CARLA client [44]. The MTMs are programmable using DVRK [45] via ROS as well. The control PC runs the *cisst-component* to interface DVRK (MTMs and the head sensor) to ROS, and also the CARLA server and client.

## 4. Experimental Protocol

The aim of the experiments was to create simulated critical conditions during autonomous car driving in LoA 3, and examine handover. For this, we created four scenarios, where the subjects had to react to an alarm given by the simulator. Fifteen participants were recruited as volunteers, all holding valid driving licenses. Prior to the experiment, we recorded their driving history, including number of years, frequency, and ADAS experience. One subject performed only one experiment. Before the simulations, every subject had one minute to practice driving in the simulator. After each scenario, subjects had to fill a questionnaire about their observations and their satisfaction.

**Figure 5:**
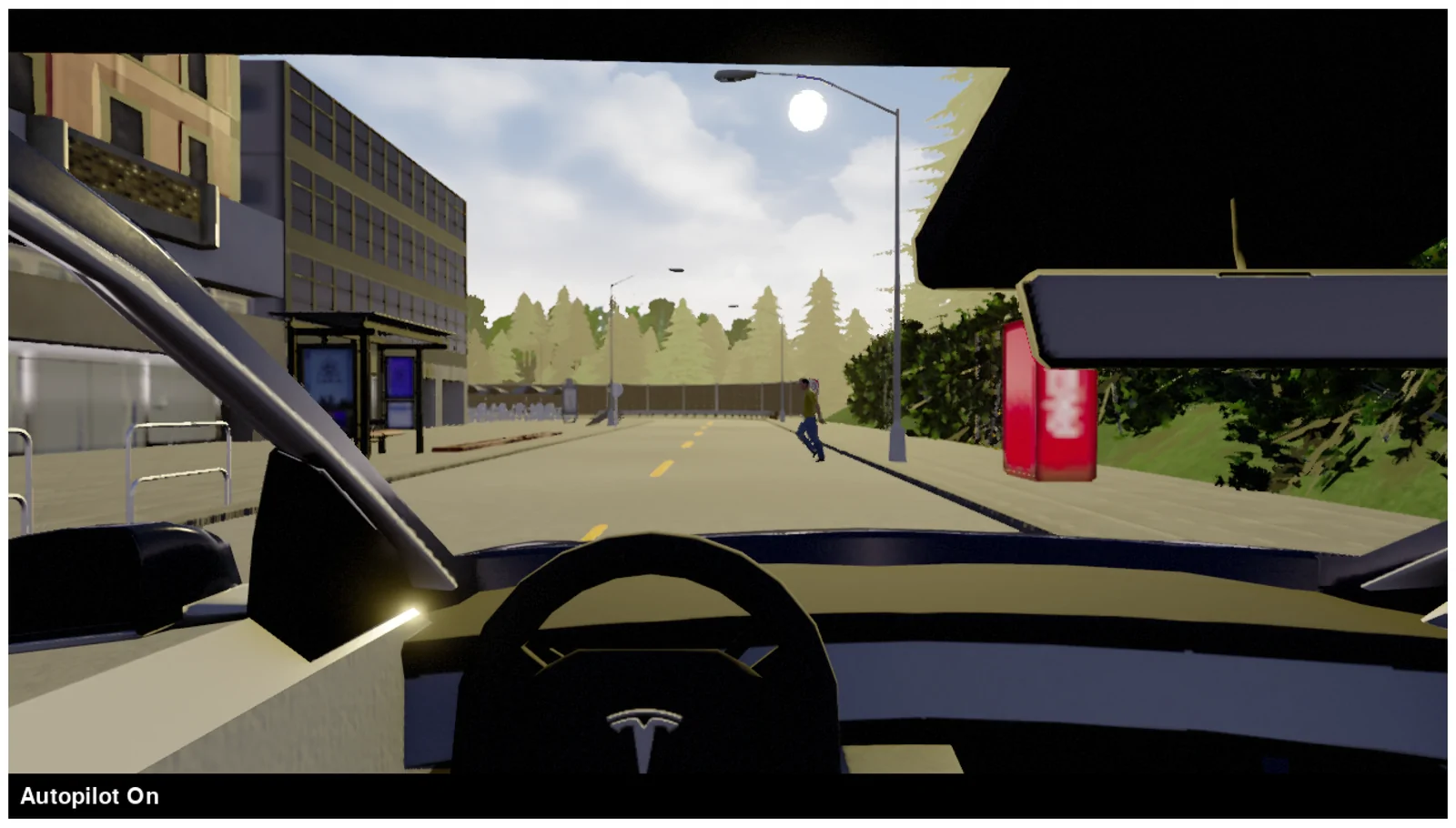
Simulation screenshot of one scenario (true alarm, no opposite traffic).

All of the scenarios started with autonomous driving. During these sessions, subjects were instructed to keep their heads out of the master console, and type a text message into a phone. This way, subjects could not pay attention to the simulated environment. The autonomous session in the scenarios took between 40 and 60 s randomly—same for each subject for comparability, but different in the scenarios for unpredictability. After the autonomous session, the system raised an emergency audio alarm and yielded the control to the human operator. The subject had to take the control from the system and solve the situation which could not be handled by the car. Subjects were also informed that unnecessary braking shall inflict penalty. All of the scenarios happened at the same location of the simulation’s map, and the weather was clear in all of the cases (Figure 5). The scenarios involved a pedestrian emergency with or without oncoming traffic. The alarm of the pedestrian was true or false. The scenarios were the combinations of the following conditions:

- **True alarm:** The pedestrian stepped in front of the car from behind a vending machine (Figure 5), close enough to hit them (alarm raised 3 s before the vehicle reaches the pedestrian).
- **False alarm:** The pedestrian was moving on the sidewalk, with safe distance from the car (alarm raised 3 seconds before the vehicle reaches the pedestrian).
- **Car arriving:** There was oncoming traffic.
- **No car arriving:** There was no oncoming traffic.

From the combinations, 4 different scenarios were compiled:

1. True alarm, no car arriving;
2. False alarm, car arriving;
3. True alarm, car arriving;
4. False alarm, no car arriving.

Before the experiment and after each scenario, subjects were asked to fill in a Google Forms (Google LLC., Mountain View, CA, USA) questionnaire about their experiences (Table 1). Before the questionnaire, they agreed to the terms of the experiment and the data were anonymous.

## 5. Results

There were 15 subjects: 13 males and 2 females, mostly young adults (ages 21–34). The subjects had never driven a car with ADAS before, except for one, who could not tell.

**Figure 6:**
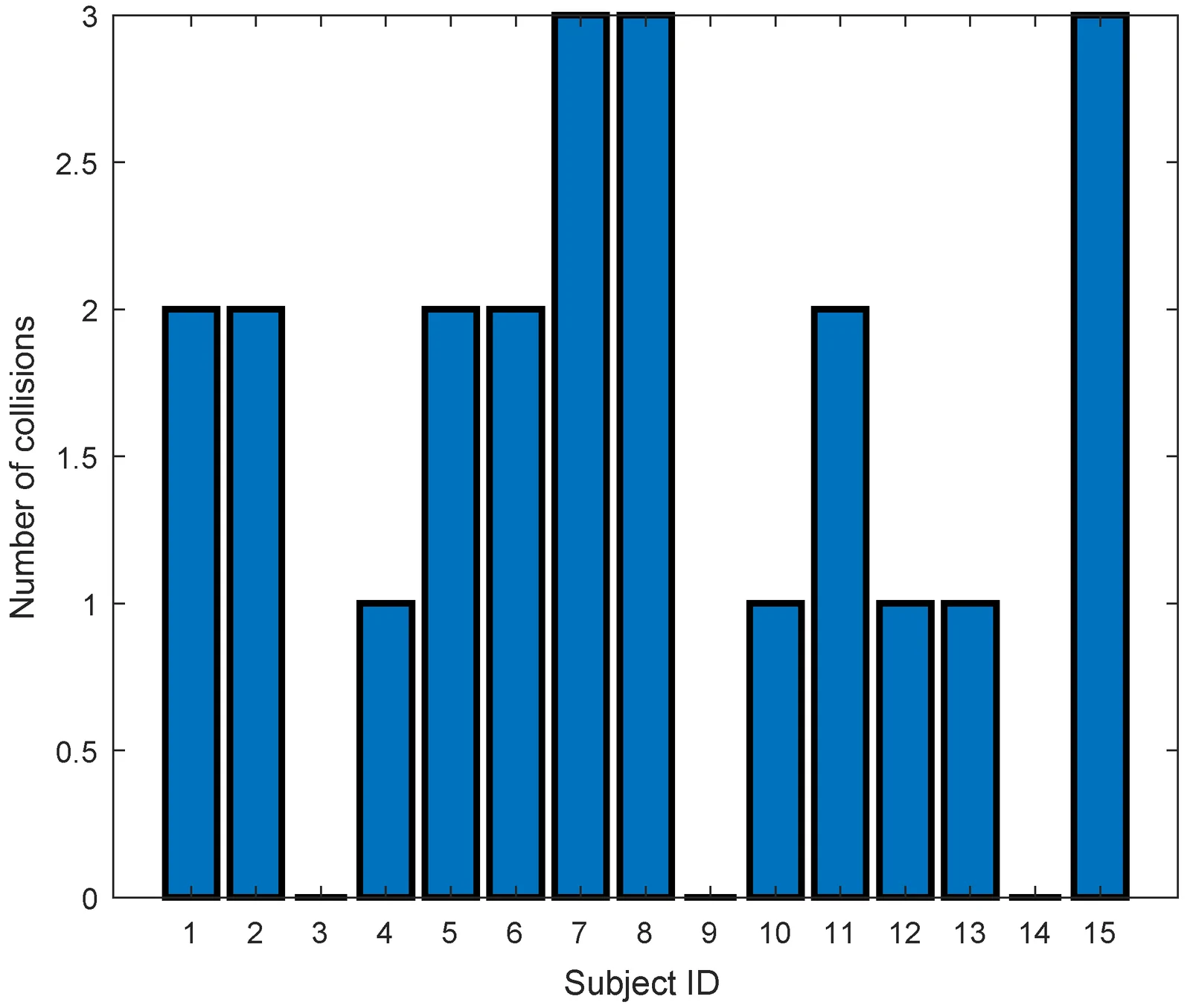
The number of scenarios with collisions for each participant.

**Figure 7:**
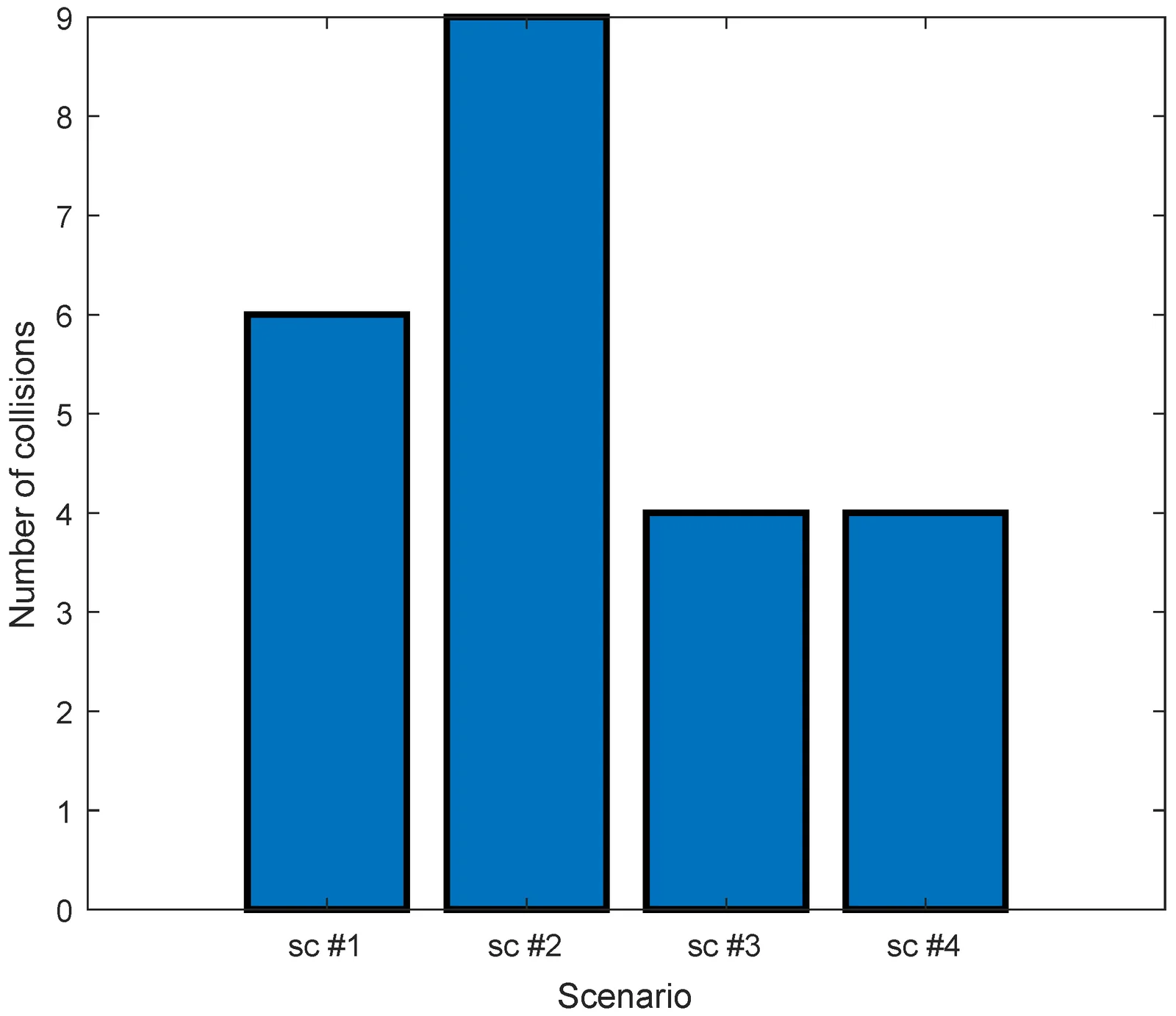
The number of collisions for each scenario.

The number of scenarios with collisions for each participant is shown in Figure 6. There were a relatively high number of collisions, and most of the time the participants collided with the curb. The number of collisions per scenarios is shown in Figure 7. The number of collisions increased during the second scenario, probably due to the car coming from the front lane, regardless of the fact that there was a false alarm. The number of collisions was smaller for the last two scenarios, which were repeated scenarios in a sense that the participants had experienced front traffic and true and false alarms as well (i.e., all the components of the scenarios), and the participants had larger SA.

**Figure 8:**
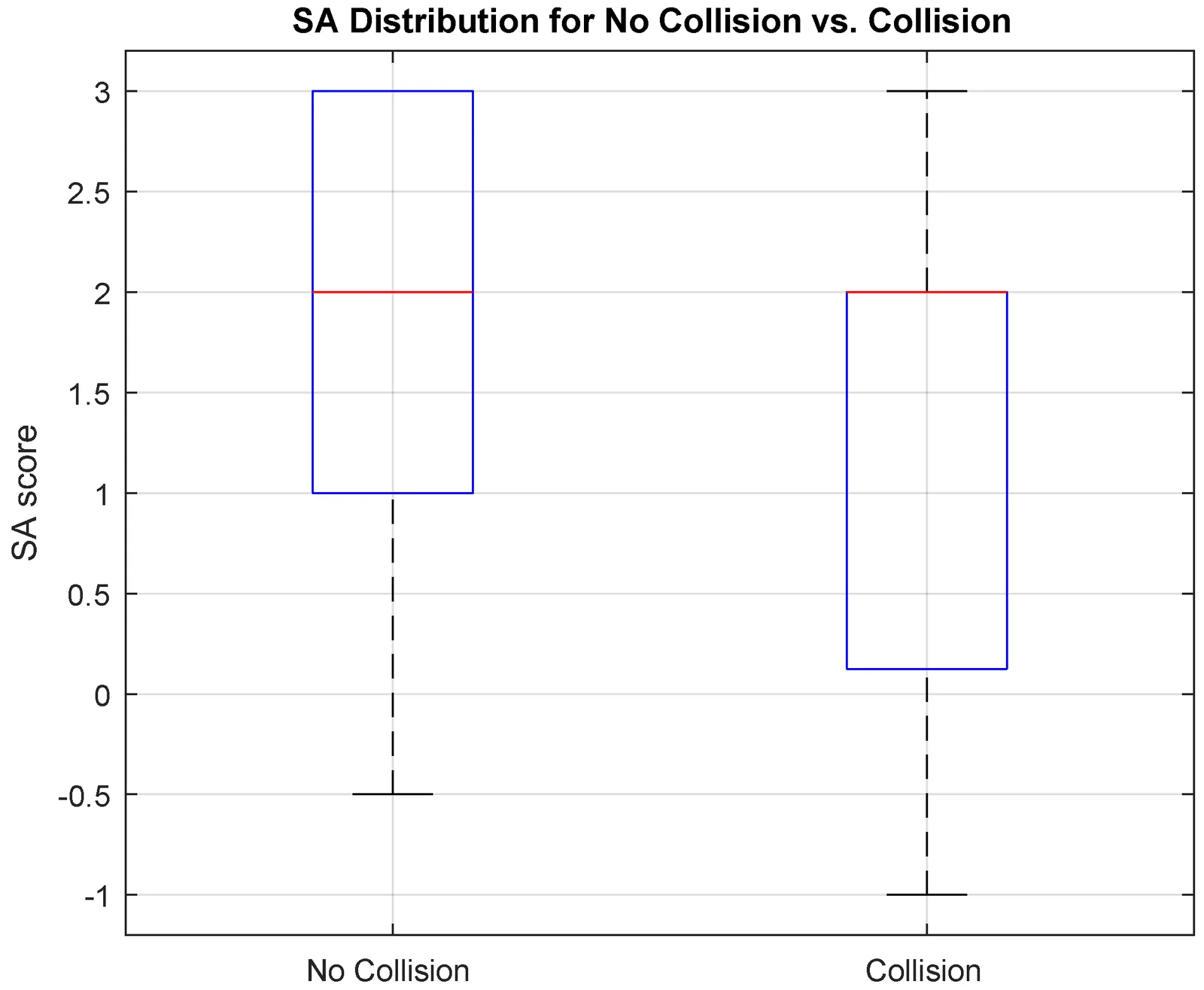
The SA score distribution of the participants in the four scenarios, with and without collision; the red line indicates the median.

The SA of the subjects was calculated based on their answers about the environment in the questionnaire. Good answers gained 1 point, wrong answers resulted in −1 point, neutral answers were 0 point. In the question about the direction of the road, the right answer was left, but straight was accepted as a correct answer with 0.5 point, since the left turn was not directly after the place of the potential accident. The mean SA for non-collision cases was 3.873, whereas for collision cases, it was lower at 2.785 (Figure 8). However, the difference was not statistically significant (*t*-test [46], p=0.078, SA values found to follow the normal distribution). The correlation between SA and collisions was analyzed using Pearson’s correlation coefficient [47]. For the whole dataset, SA and collisions showed weak negative correlation, but the result was not statistically significant (correlation coefficient r=−0.229, p=0.078). The four scenarios were also analyzed separately; significant strong negative correlation was found between SA and collisions in *scenario 3* (r=−0.596, p=0.019) and *scenario 4* (r=−0.551, p=0.033).

The post-scenario questionnaire results further highlight gaps in awareness:

- **Scenario 1** (true alarm, no oncoming traffic):
  - 80% correctly identified the pedestrian as the cause of the emergency;
  - Only 40% remembered the pedestrian’s pants color;
  - 53.3% correctly identified the road’s leftward turn.
- **Scenario 2** (false alarm, oncoming traffic):
  - 73.3% recognized that the emergency was due to the automation system;
  - 86.7% correctly recalled that a forest was on the right;
  - Only 26.7% identified the correct speed limit.
- **Scenario 3** (true alarm, oncoming traffic):
  - 80% correctly identified the pedestrian as the cause of the emergency;
  - Only 33.3% placed correctly the bus stop location;
  - 73.3% realized there was no pedestrian crosswalk.
- **Scenario 4** (false alarm, no oncoming traffic):
  - 60% identified the automation system as the cause of the emergency;
  - 93.3% correctly noted the absence of oncoming traffic;
  - 93.3% correctly identified houses on the left.

**Figure 9:**
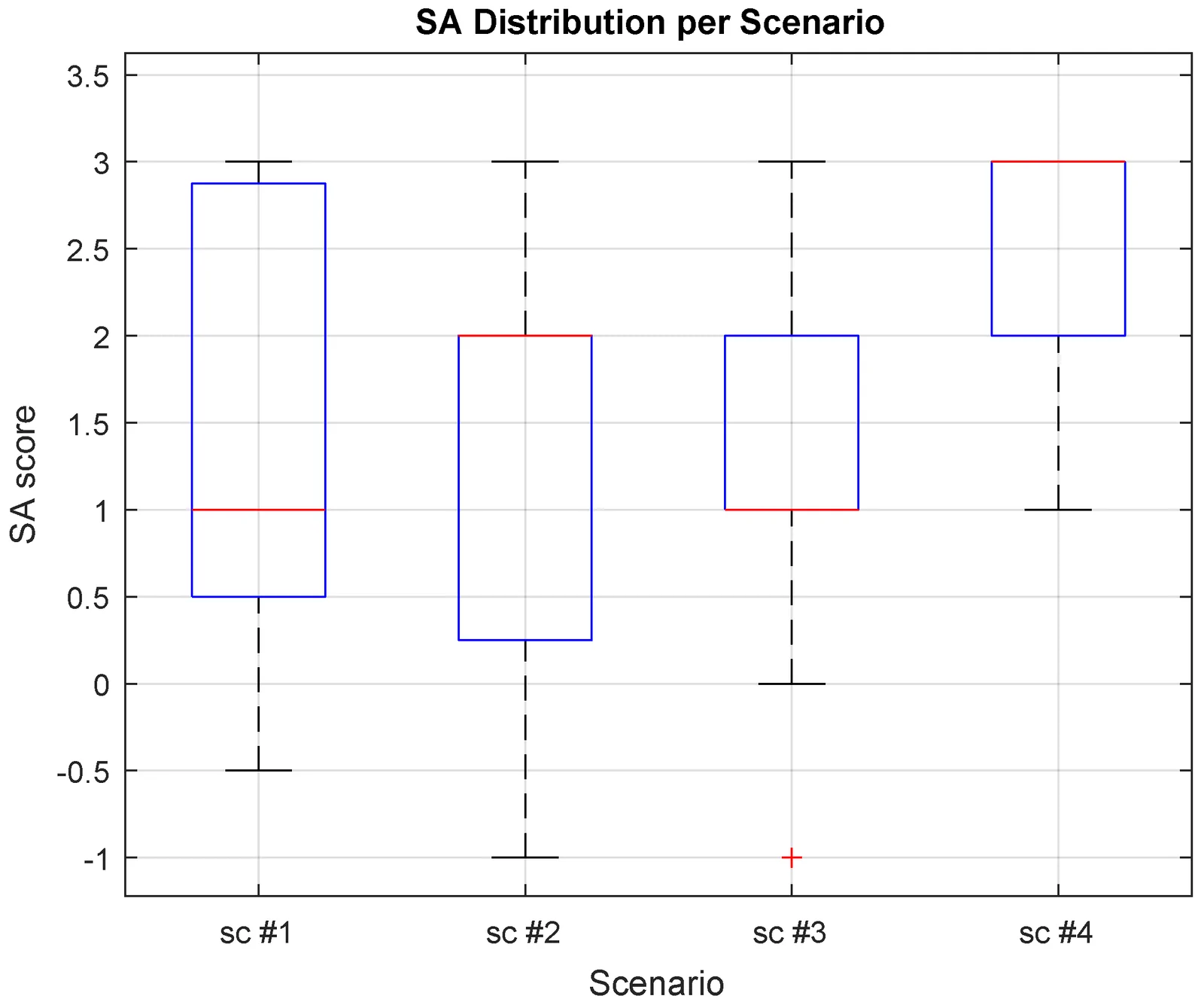
The SA score of the participants in the four scenarios; the red line indicates the median.

These findings indicate progressive improvement in SA but also highlight areas where SA remained inconsistent, such as recognizing road infrastructure details. Figure 9 illustrates the SA scores across the four scenarios, demonstrating the mentioned increasing trend. This learning effect was evaluated using linear regression analysis [48] and Cohen’s *d* [49]. The SA score was found to increase by 0.27 per scenario (p=0.044, significant), but the effect size was small (Cohen’s d=0.467). This suggests that the subjects’ ability to assess and respond to the environment improved with exposure to the task. Such improvements could be attributed to increased familiarity with the task, reduced cognitive load, or greater confidence in decision making as subjects progress through the experiment.

The observed increase in SA across scenarios, along with the correlation between SA and collisions emerging only in the final two scenarios, suggests that a minimum threshold of SA may be necessary for effectively managing emergency situations. Given the nature of the scenario, this threshold is likely at SA Level 2. However, quantifying SA levels based on questionnaire responses is not straightforward. A potential approach to determine this threshold is to identify the SA level at which the number of collisions decreases significantly. This could be achieved through a repeated study with a larger sample size to enhance statistical reliability.

**Figure 10:**
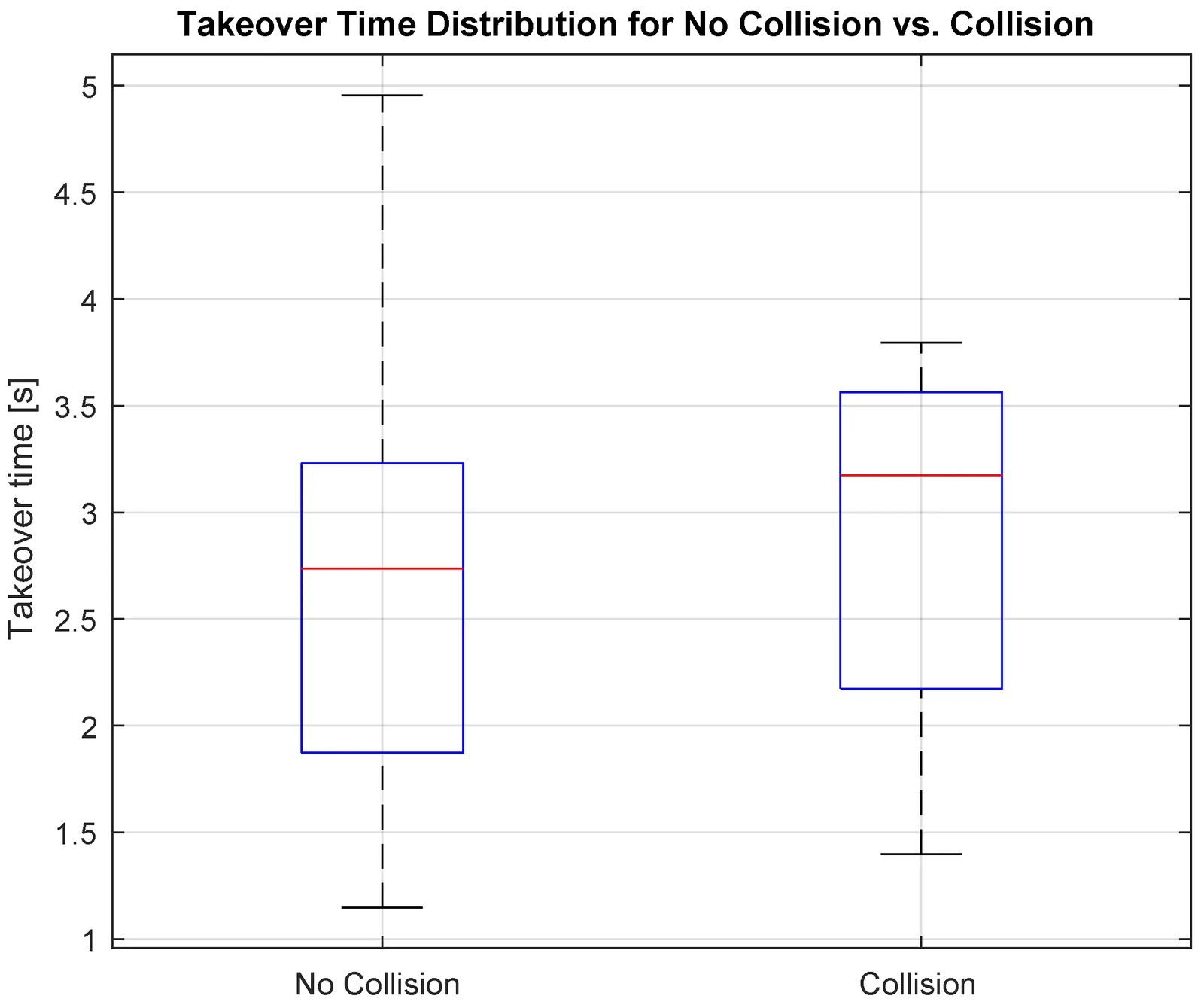
The takeover times of the 15 subjects during the four scenarios, with and without collision; the red line indicates the median.

**Figure 11:**
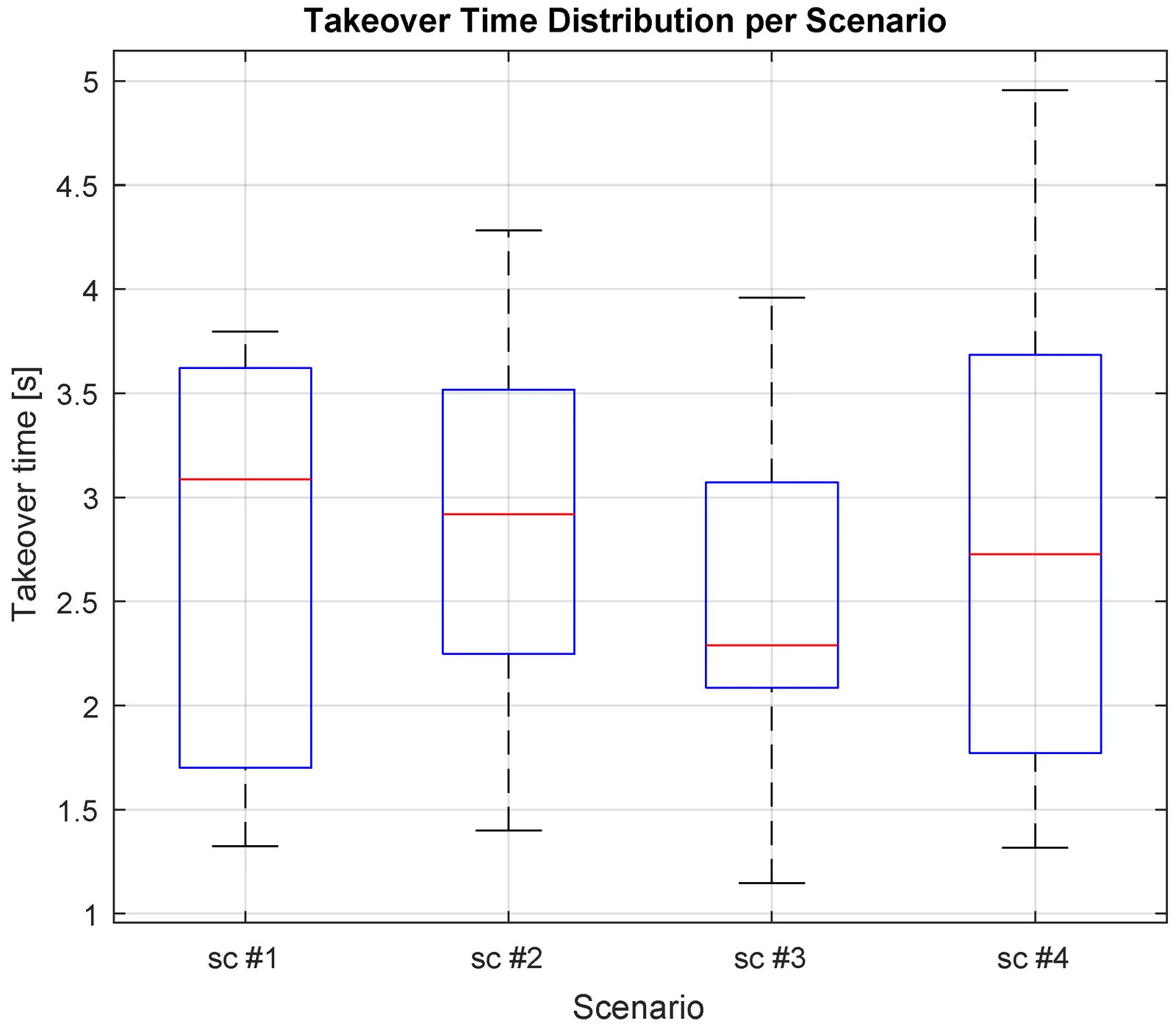
The takeover times of the 15 subjects during the four scenarios; the red line indicates the median.

Figure 10 and Figure 11 show the takeover times for scenarios with and without collision and for each scenario across all participants, respectively. The mean takeover time for collision cases was 2.82 s, while for non-collision cases, it was slightly lower at 2.7 s. However, no significant correlation was revealed between takeover time and collision occurrence (r=0.093, p=0.489) or between takeover time and SA (r=0.139, p=0.297) by statistical analysis. Moreover, as takeover time did not correlate with the number of collisions, while the SA score did, this might suggest that Level 2 SA of the driver (comprehension of the current situation) has a decisive role in handover performance, while Level 1 SA (perception of the environment) itself may not be enough for a proper reaction.

Additionally, the analysis of the effect of practice showed no significant impact on takeover time. These findings suggest that while SA increases and the number of collisions decreases over repeated scenarios, takeover time remains relatively stable. This implies that repetition does not necessarily decrease takeover time, but it improves SA which decreases the chance of collisions [50].

**Figure 12:**
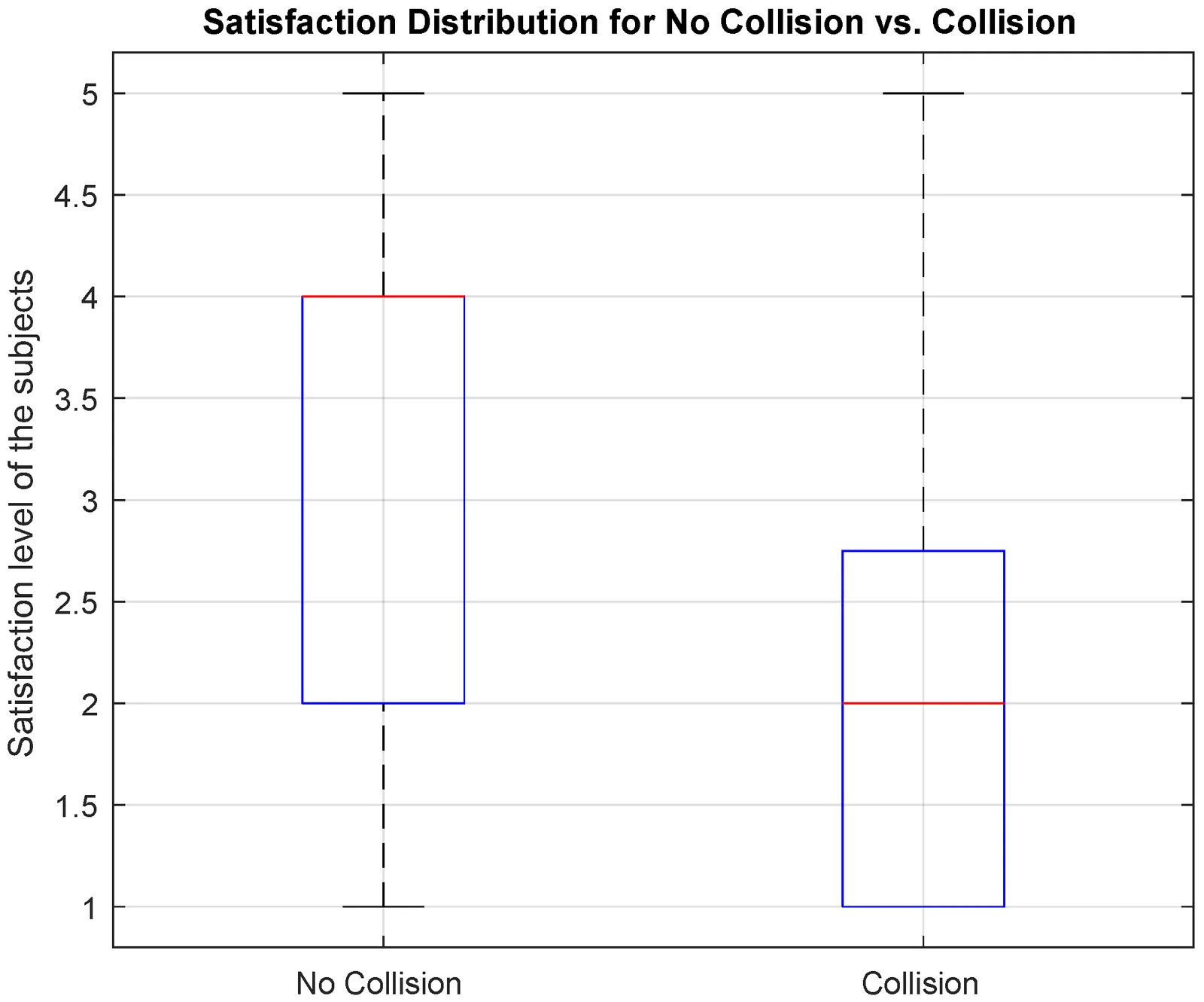
The satisfaction of the 15 subjects during the four scenarios, with and without collision; the red line indicates the median.

**Figure 13:**
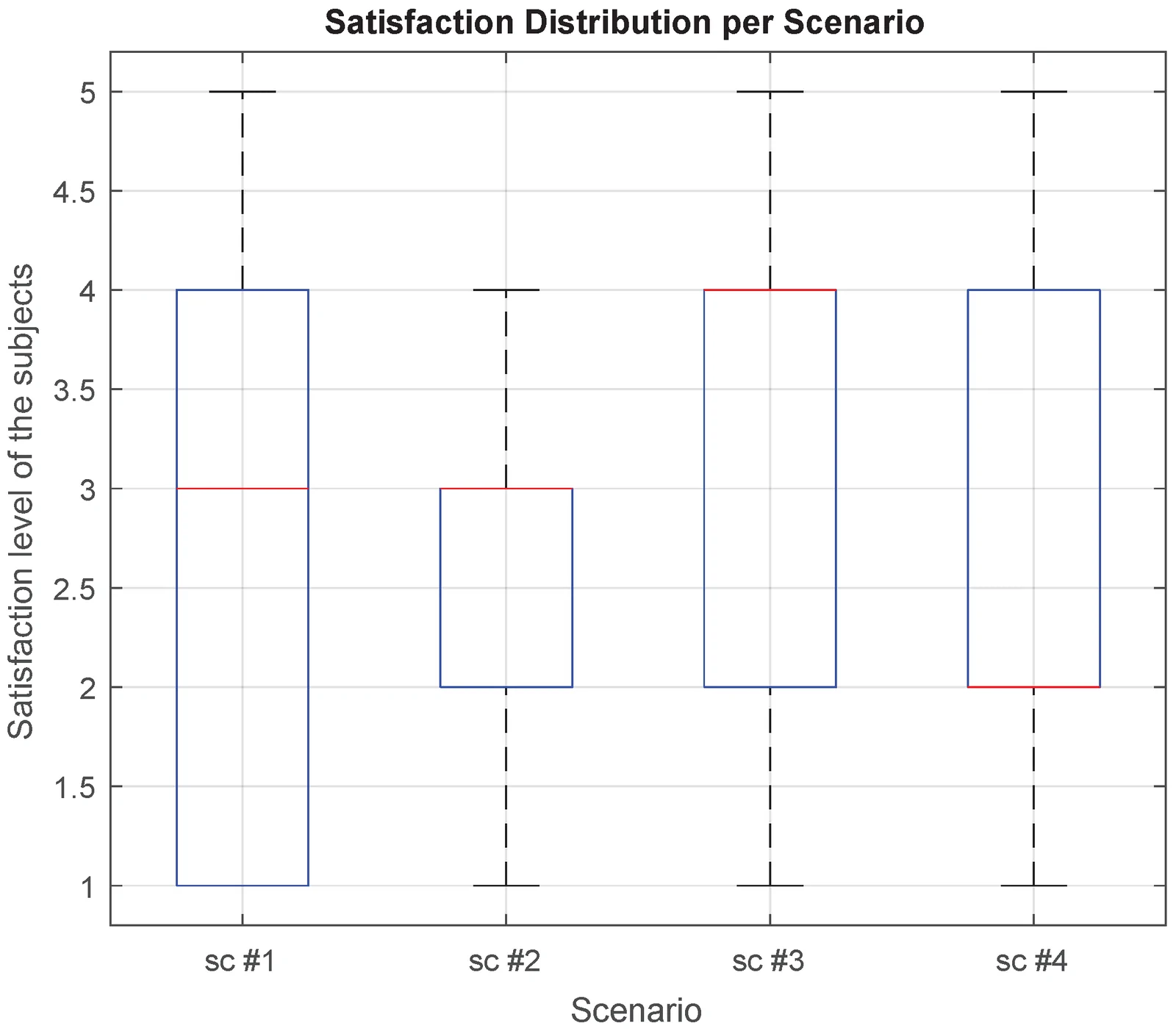
The satisfaction distribution of the participants in the four scenarios, with and without collision; the red line indicates the median.

The satisfaction level of the subjects, based on the questionnaire, is shown for scenarios with and without collision and for each scenario across all participants in Figure 12 and Figure 13. The mean satisfaction level for the cases with no collision is 3.35, while the mean satisfaction level at the cases with collision is 1.93. The results imply strong, statistically significant negative correlation between satisfaction and collisions (r=−0.512, p=0.000029), thus collision decreased the satisfaction of the participants. Also, the satisfaction did not improve with the scenarios, so gaining SA does not improve satisfaction.

The potential correlation between driving experience and key performance metrics, including SA, takeover time, and the number of collisions, was analyzed. The results indicated no statistically significant relationships, with correlation values of (r=0.0445, p=0.875) for SA, (r=0.185, p=0.51) for takeover time, and (r=0.0488, p=0.863) for collisions. One possible explanation is that automation levels the playing field, as all participants were disengaged from the driving task during autonomous operation, reducing any potential advantage of prior driving experience. Additionally, handover performance may depend more on automation-specific learning and cognitive adaptation rather than traditional driving skills [51].

## 6. Conclusions

In this article, the quantitative analysis of SA during LoA 3 handover scenarios was presented. We reviewed the current state of the development of autonomous driving from the aspect of safety, and that how SA might influence the driver’s handover performance. An experimental study was compiled using the da Vinci Surgical System and the CARLA driving simulator involving 15 test subjects, where the subjects had to perform emergency handover tasks during autonomous driving.

The results support the notion that higher SA improves task performance in handover situations. It was shown that the takeover time of the participants did not significantly decrease across the four successive scenarios. In contrast, the SA scores, derived from the questionnaire responses, showed an increasing trend, interpretable as a learning curve, while the number of collisions decreased. These results suggest that the success of the handover maneuver is strongly influenced by the driver’s SA. Additionally, the analysis revealed no correlation between takeover time and the number of collisions, while the SA score showed a partial correlation (notably in the last two scenarios). This implies that Level 2 SA (comprehension of the current situation) plays a crucial role in handover performance, while Level 1 SA alone may not be sufficient for an appropriate response.

These findings underline the crucial role that SA—particularly the higher-level cognitive processes associated with understanding and predicting the unfolding situation—plays in LoA 3 handover scenarios. The results suggest that training drivers to develop and maintain this level of awareness could enhance their ability to safely transition control in emergency situations.

It is important to acknowledge the potential psychological factors that could influence the performance and satisfaction ratings of participants. Anticipation and stress, particularly during critical alarm scenarios, could have affected participants’ behavior and reaction times. The awareness of the simulated nature of the experiment might have mitigated the emotional intensity compared to real-world situations, but nonetheless, could still have impacted engagement levels and response selection. Additionally, psychological influences such as confidence in their abilities and perceived control over the situation may have affected the participants’ ratings of satisfaction. Future studies could further explore these factors by incorporating more in-depth psychological assessments and subjective well-being measures during the experiment.

In our future work, the number of test subjects shall be increased, to be able to compile more certain statistical results. Also, the experiments on a control group with continuous attention to the road might also better certify the results obtained.

The supporting software platform developed for this study has been released as an open-source project and is available at: https://github.com/ABC-iRobotics/dvrk_carla (accessed on 29 May 2025).

## Figures and Tables

**Table 1:** Questions of the questionnaire the subjects were asked to fill in during certain points of the experiment.

Before the Experiment	Scenario 1	Scenario 2	Scenario 3	Scenario 4
Age; Gender; When did they gain a driving license?; How frequently do they drive?; Did they ever drive a car with ADAS?	How lifelike were the simulator and the scenario?; Evaluate their own reaction on a scale 1–5; Who/what caused the emergency?; What was the color of the pedestrian’s pants?; After the alarm, what was the direction of the road?	Evaluate their own reaction on a scale 1–5; Who/what caused the emergency?; Where was the bus station during the emergency (what side of the road)?; Why did they not stop at the pedestrian crossing?	Evaluate their own reaction on a scale 1–5; Who/what caused the emergency?; What was on the right side of the road during the emergency?; After the emergency, what could be your maximum speed?	How lifelike were the simulator and the scenario?; Evaluate their own reaction on a scale 1–5; Who/what caused the emergency?; Was there oncoming traffic?; What was on the left side of the road during the emergency?

## Data Availability

The data presented in this study are openly available in GitHub: https://github.com/ABC-iRobotics/dvrk_carla.

## Abbreviations

The following abbreviations are used in this manuscript:

ADAS	Advanced driver assistance systems
DVRK	Da Vinci Research Kit
HMI	Human–machine interface
LoA	Level of autonomy
MTM	Master tool manipulator
RAMIS	Robot-assisted minimally invasive surgery
ROS	Robot Operating System
SA	Situation awareness
SAE	Society of Automotive Engineers
SAGAT	Situation awareness global assessment technique
SART	Situational awareness rating technique
